# Supplementary material for: Fresnel Magnetic Imaging of Ultrasmall Skyrmion Lattices
Source: Adv Sci (Weinh). 2025 Dec 3;13(4):e09786. doi: 10.1002/advs.202509786 (PMC12822451; doi:10.1002/advs.202509786)
Supplement: Supplementary file 1 — Supporting Information [file ADVS-13-e09786-s001.pdf]

Supporting Information

Fresnel magnetic imaging of ultrasmall skyrmion lattice

Yongsen Zhang, Wei Liu, Meng Shi, Yaodong Wu*, Jialiang Jiang, Sheng Qiu, Huanhuan Zhang, Hui Han, Mingliang Tian, Haifeng Du, Shouguo Wang* and Jin Tang*

Y. Zhang, S. Wang

Anhui Provincial Key Laboratory of Magnetic Functional Materials and Devices, School of Materials Science and Engineering, Anhui University, Hefei 230601, China

Email: sgwang@ahu.edu.cn

W. Liu, H. Han

Institutes of Physical Science and Information Technology, Anhui University, Hefei 230601, China

M. Shi, S. Qiu, M. Tian, H. Du

Anhui Provincial Key Laboratory of Low-Energy Quantum Materials and Devices, High Magnetic Field Laboratory, HFIPS, Chinese Academy of Sciences, Hefei, Anhui 230031, China

Y. Wu

School of Physics and Materials Engineering, Hefei Normal University, Hefei, 230601, China

Email: wuyaodong@hfnu.edu.cn

J. Jiang, H. Zhang, J. Tang

School of Physics and Optoelectronic Engineering, Anhui University, Hefei, 230601, China

Email: jintang@ahu.edu.cn

This Supporting Information contains the following sections.

Figure S1 -- The fundamental magnetic properties of $Fe_{0.5}Mn_{0.5}Ge$.

Figure S2 -- Comparative analysis of Transport of Intensity Equation (TIE) solutions under varying q_0 parameters

Figure S3 -- Fresnel imaging of individual 15-nm skyrmions at various defocus values.

Figure S4 -- Fresnel images of skyrmions in regions of different thicknesses within the same sample, captured at various defocus values.

Figure S5 -- Simulated Fresnel images of 15 nm skyrmions in regions with thicknesses of 75 nm and 150 nm at various defocus values.

Figure S6 -- The effect of external magnetic field strength on Δf.

Figure S7 -- Effect of the DMI sign on contrast reversal in skyrmion lattices of different sizes.

Figure S8 -- Simulated Fresnel images of skyrmions with different diameters at various defocus values.

Figure S9 -- Evolution of skyrmion lattice size with temperature.

Figure S10 -- Fresnel images of the skyrmion lattice in FeGe under varying defocus conditions in the experiment.

Figure S11 -- Simulated Fresnel images of skyrmions with diameters of 10 nm and 15 nm within the same sample at different defocus values.

Figure S12 -- Fresnel contrast intensity as a function of defocus for a simulated skyrmion lattice with $\lambda_{sk} \approx$ 15 nm and 70 nm.

Figure S13 -- Effect of aberration settings (zero vs. non-zero) on Δf.

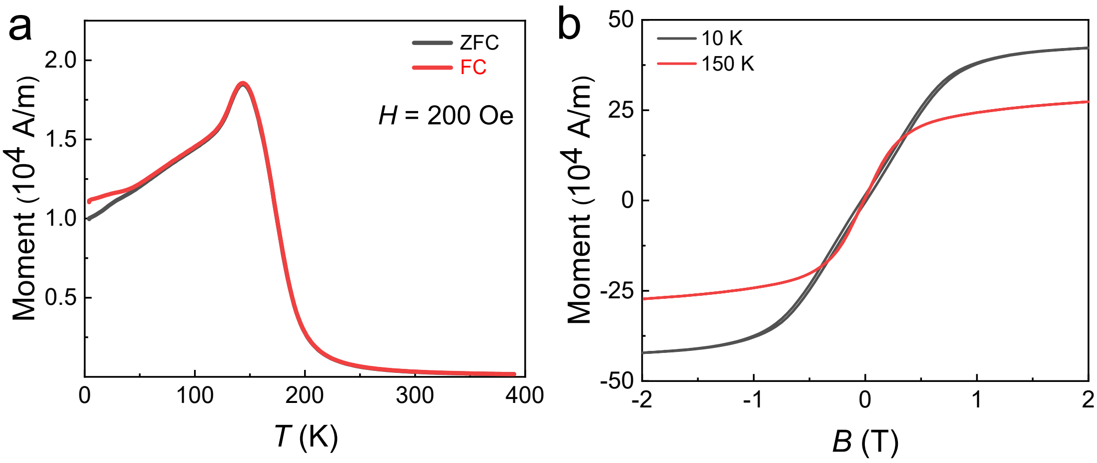

Figure S1. The fundamental magnetic properties of $Fe_{0.5}Mn_{0.5}Ge$. **a**, Temperature dependence of magnetization curves measured under an external field of 200 Oe using zero field cooling (ZFC) and field cooling (FC) protocols. **b**, Field dependence of magnetization at 10 K (black line) and 150 K (red line).

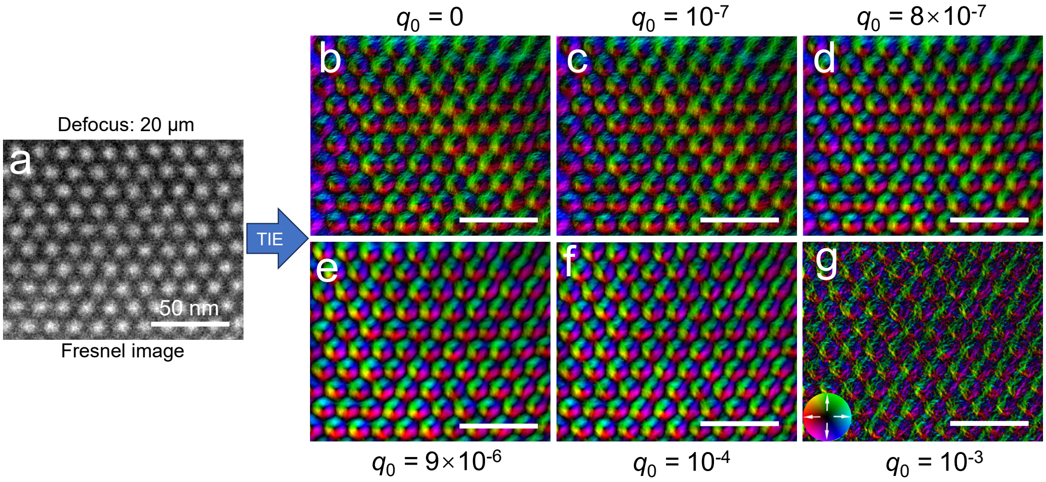

Figure S2. Comparative analysis of Transport of Intensity Equation (TIE) solutions under varying q_0 parameters

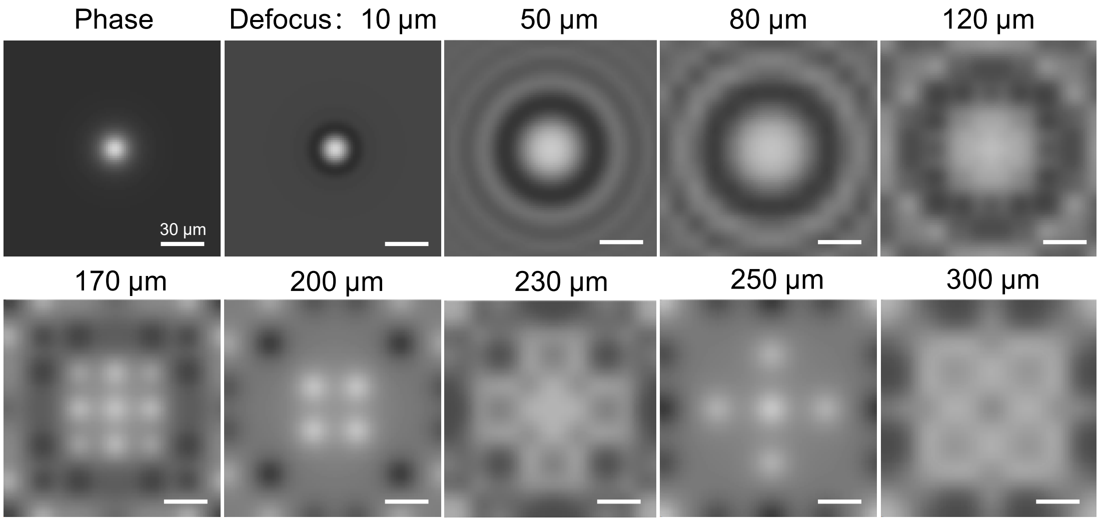

Figure S3. Fresnel imaging of individual 15-nm skyrmions at various defocus values.

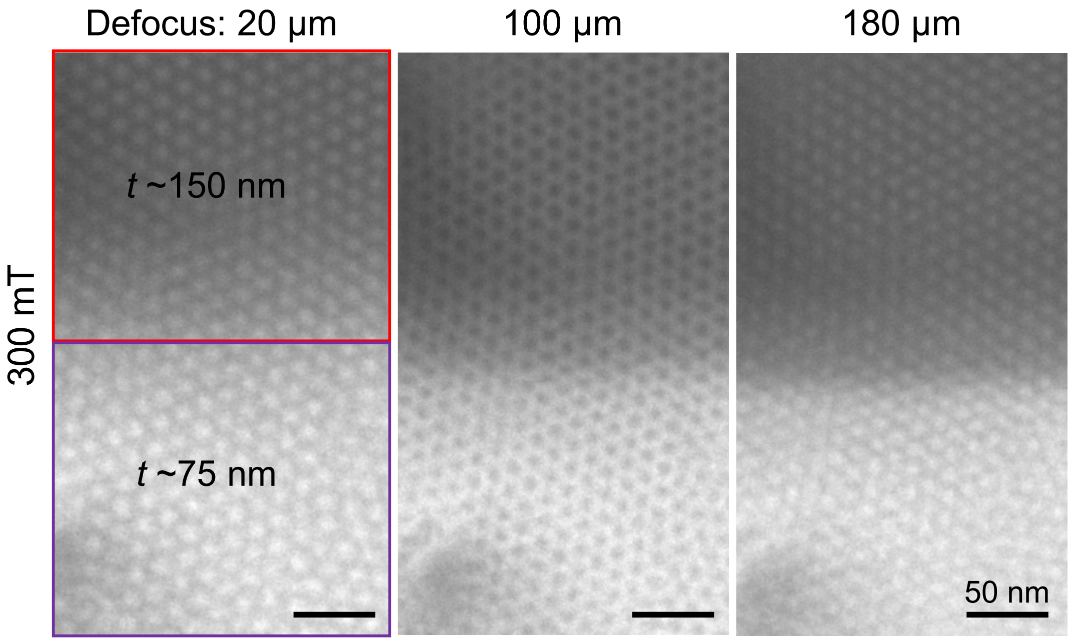

Figure S4. Fresnel images of skyrmions in regions of different thicknesses within the same sample, captured at various defocus values. The red-boxed rectangular region has a thickness of 150 nm, while the purple-boxed rectangular region has a thickness of 75 nm. An external magnetic field of 300 mT was applied.

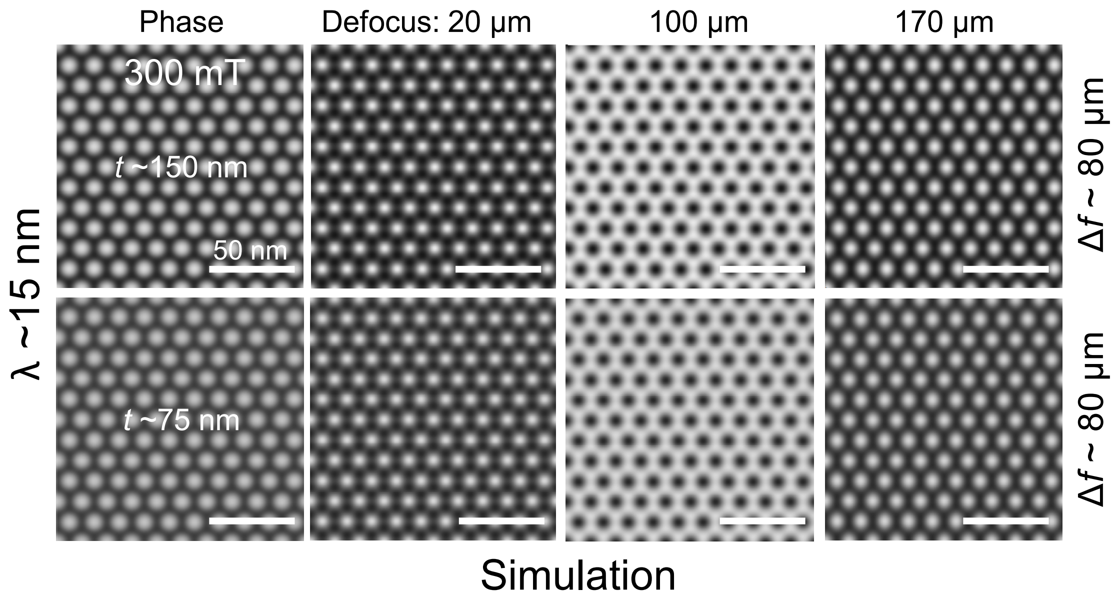

Figure S5. Simulated Fresnel images of 15 nm skyrmions in regions with thicknesses of 75 nm and 150 nm at various defocus values. Δf represents the defocus difference with inverted black-and-white contrast.

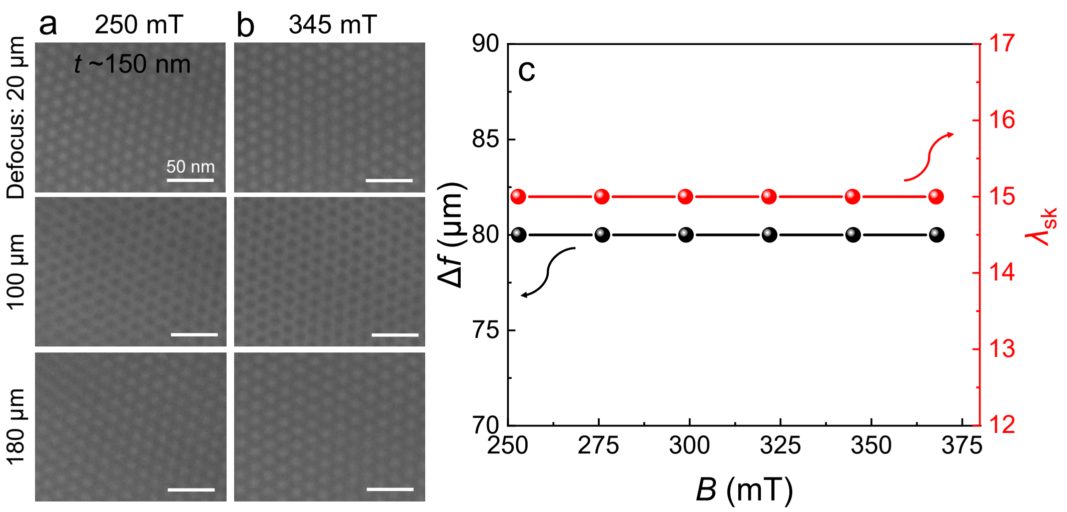

Figure S6. The effect of external magnetic field strength on Δ*f*. **a**, and **b**, Fresnel images at various defocus values under B = 250 mT and 345 mT. **c**, The function of Δ*f* variation with the magnetic field. Δ*f* represents the defocus difference with inverted black-and-white contrast.

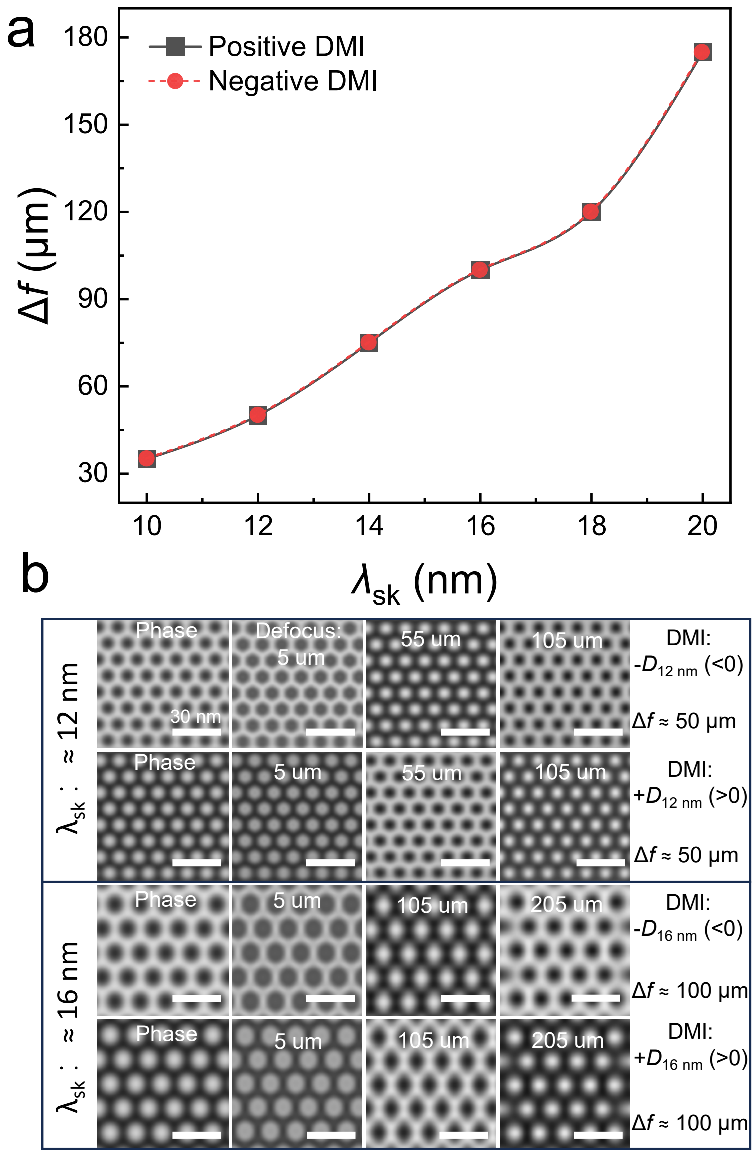

Figure S7. a, Effect of the DMI sign on contrast reversal in skyrmion lattices of different sizes. **b,** Fresnel contrast of the corresponding skyrmion lattices under different defocus values, for λ_{sk} of 12 and 16 nm with the DMI sign alternated.

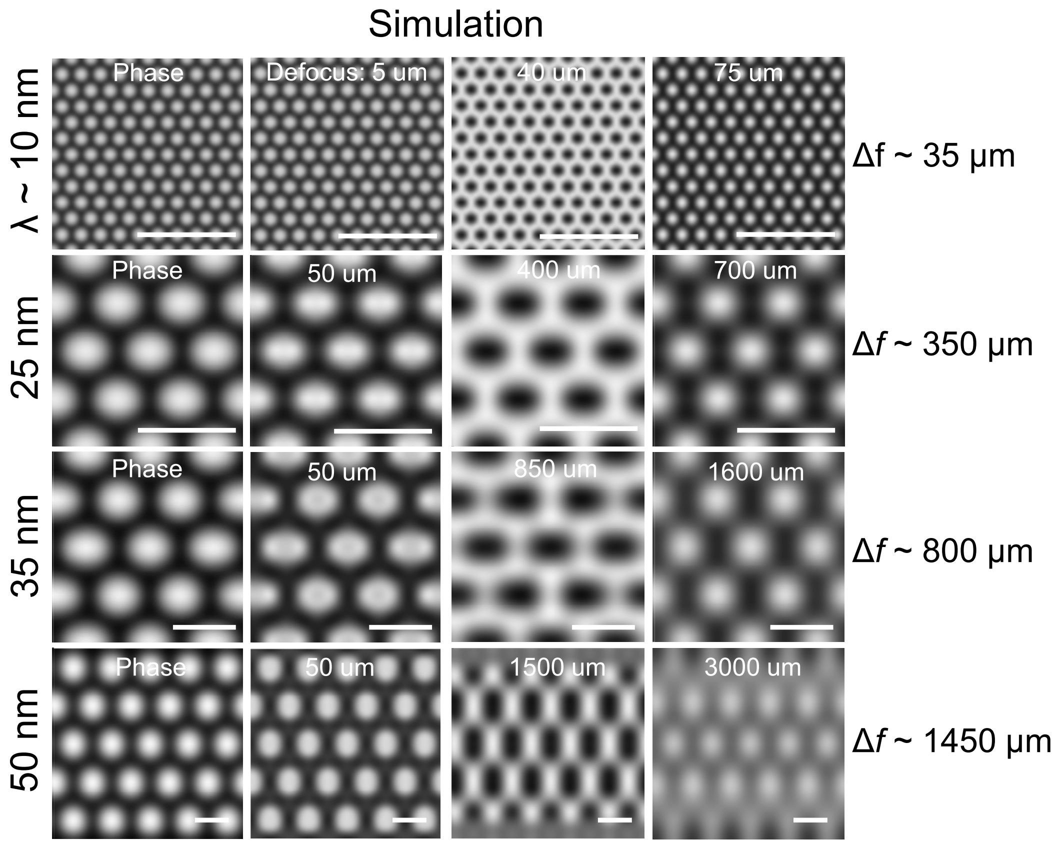

Figure S8. Simulated Fresnel images of skyrmions with different diameters at various defocus values. Δf represents the defocus difference with inverted black-and-white contrast. B = 300 mT. Scale bar, 50 nm.

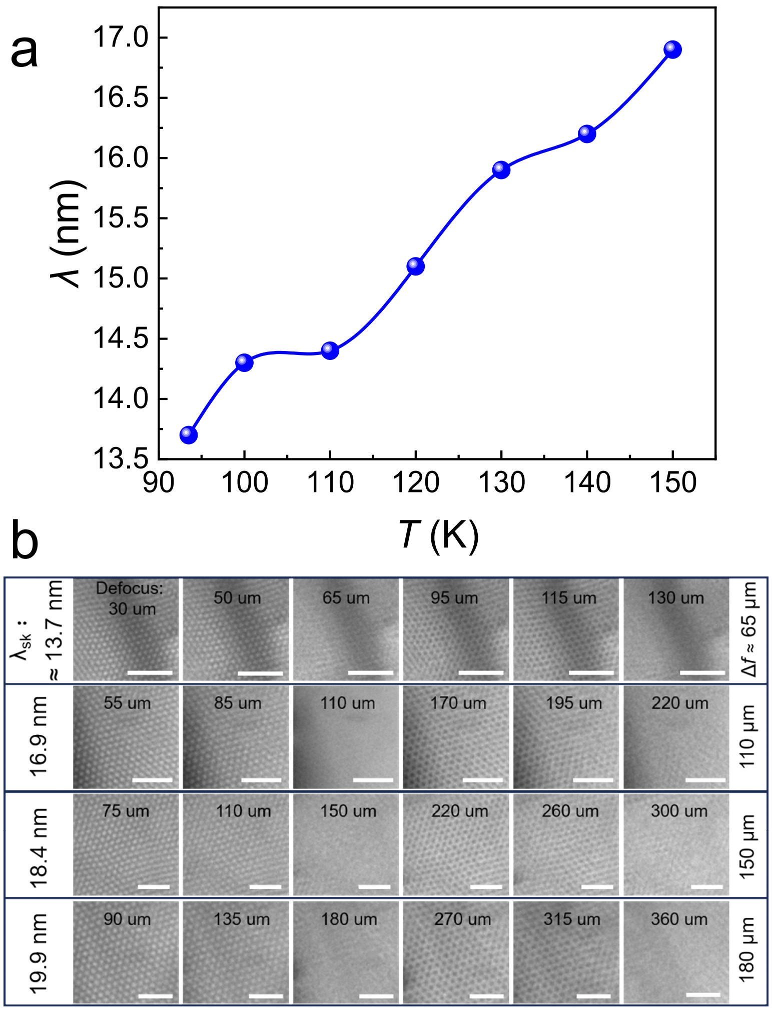

Figure S9. a, Evolution of skyrmion lattice size with temperature. **b,** Dependence of Fresnel contrast on defocus value for skyrmion lattices of various sizes. Scale bar, 100 nm.

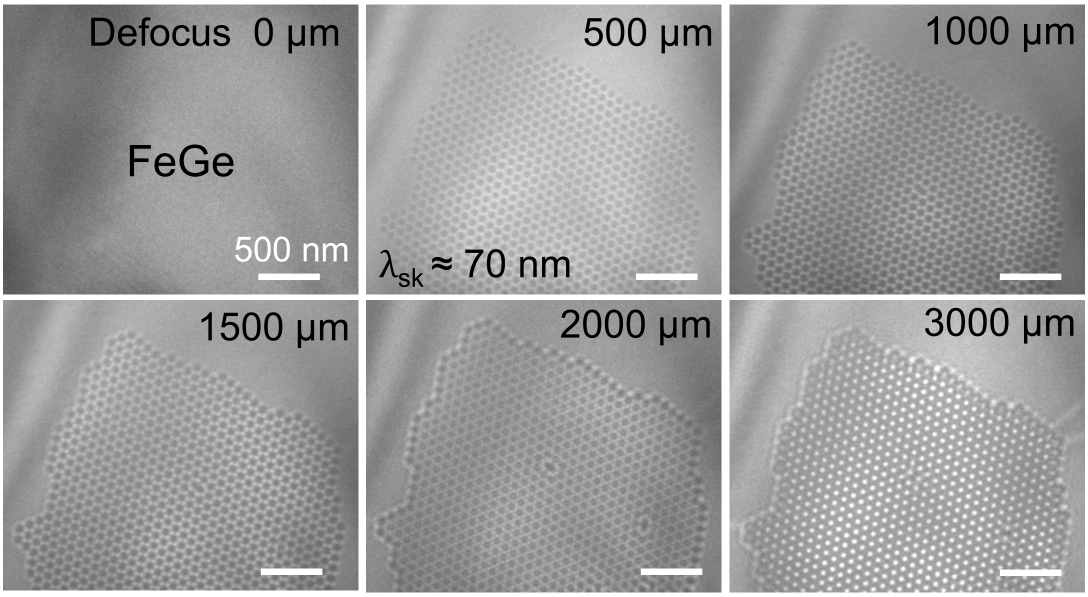

Figure S10. Fresnel images of the skyrmion lattice in FeGe under varying defocus conditions in the experiment. External magnetic field B = 100 mT. Temperature T = 240 K.

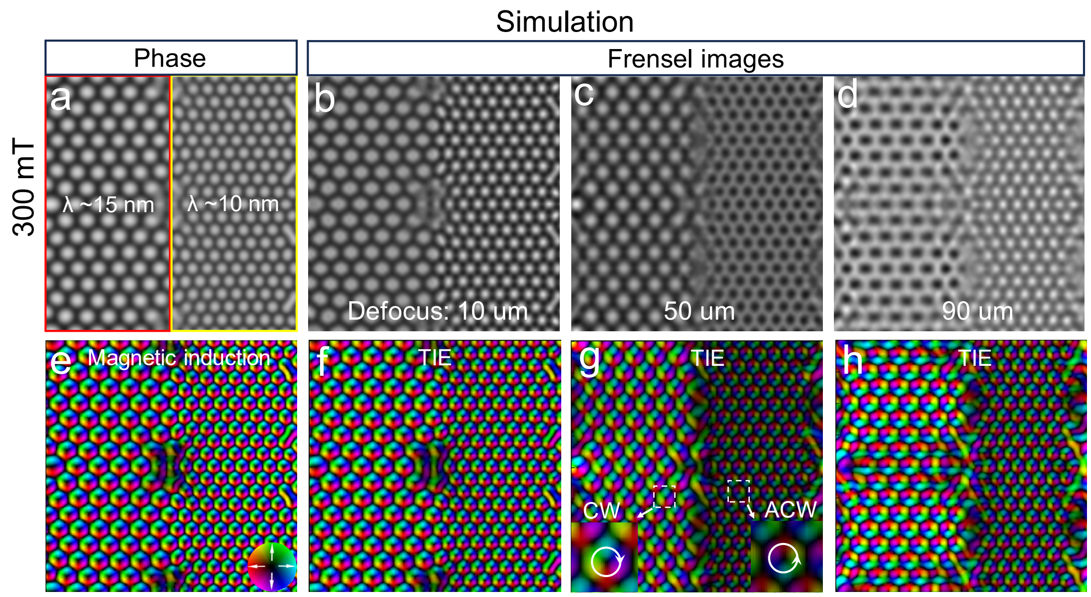

Figure S11. Simulated Fresnel images of skyrmions with diameters of 10 nm and 15 nm within the same sample at different defocus values. **a**, The magnetic phase map. **b-d**, Simulated Fresnel images at defocus values of 10 µm, 50 µm, and 100 µm. **e**, Magetic induction derived from the analysis of **a**. **f-h**, The TIE analysis corresponding to the Fresnel images in **b-d**, Red-boxed rectangular region: skyrmion diameter λ_{sk} = 15 nm; Yellow-boxed rectangular region: skyrmion diameter λ_{sk} = 10 nm. B = 300 mT.

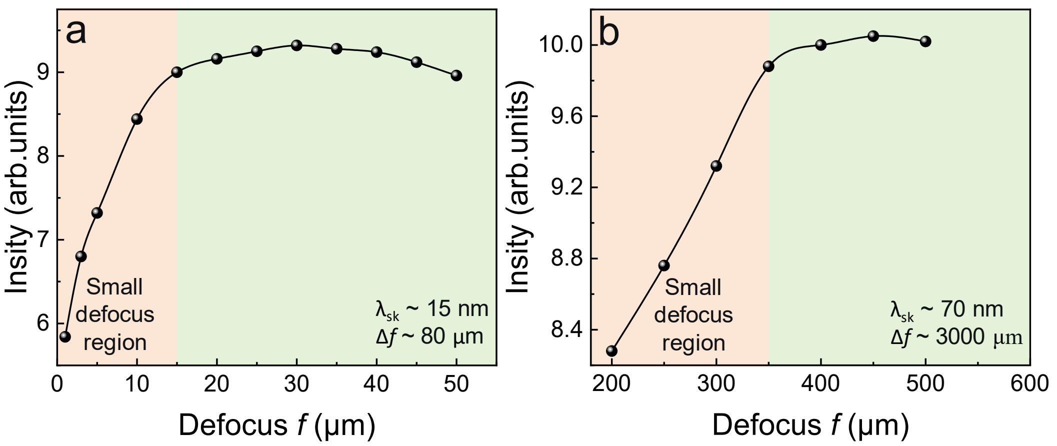

Figure S12. Fresnel contrast intensity as a function of defocus for a simulated skyrmion lattice with $\lambda_{sk} \approx 15$ nm (a) and 70 nm (b). The pink area corresponds to the small-defocus region.

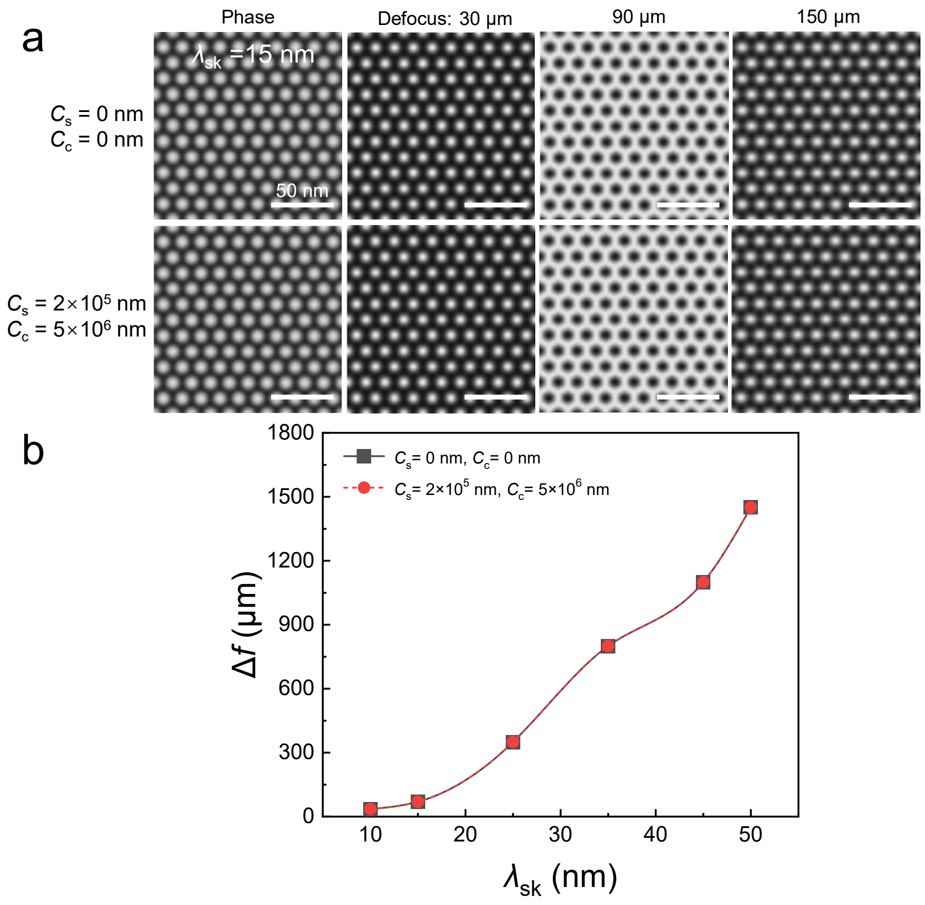

Figure S13. a, Fresnel images simulated by Pylorentz under aberration-free and aberration-present conditions. **b,** Effect of aberration settings (zero vs. non-zero) on Δf.
